# Clinical profile of children under 5 years of age with rotavirus diarrhoea in a hospital setting in Kisangani, DRC, after the introduction of the rotavirus vaccine, a cross-sectional study

**DOI:** 10.1186/s12887-023-04022-0

**Published:** 2023-04-24

**Authors:** Didier Gbebangi-Manzemu, Véronique Muyobela Kampunzu, Hortense Malikidogo Vanzwa, Mupenzi Mumbere, Gaspard Mande Bukaka, Bibi Batoko Likele, Emmanuel Tebandite Kasai, Benoit Mbiya Mukinayi, Serge Tonen-Wolyec, Nestor Ngbonda Dauly, Jean Pierre Alworong’a Opara

**Affiliations:** 1 Department of Paediatrics, Faculty of Medicine and Pharmacy, University of Kisangani, Kisangani, Democratic Republic of the Congo; 2 Department of Paediatrics, Faculty of Medicine, Catholic University of Graben, Butembo, Democratic Republic of the Congo; 3 Department of Paediatrics, Faculty of Medicine, University of Mbujimayi, Mbuji-Mayi, Democratic Republic of the Congo; 4 Department of Internal Medicine, Faculty of Medicine, University of Bunia, Bunia, Democratic Republic of the Congo

**Keywords:** Acute diarrhoea, Children under 5, Rotavirus, Kisangani

## Abstract

**Background:**

The Democratic Republic of the Congo (DRC) is one of the countries with the highest rotavirus mortality rate in the world. The aim of this study was to describe the clinical features of rotavirus infection after the introduction of rotavirus vaccination of children in the city of Kisangani, DRC.

**Methods:**

We conducted a cross-sectional study of acute diarrhoea in children under 5 years of age admitted to 4 hospitals in Kisangani, DRC. Rotavirus was detected in children’s stools by an immuno-chromatographic antigenic rapid diagnostic test.

**Results:**

A total of 165 children under 5 years of age were included in the study. We obtained 59 cases of rotavirus infection, or 36% CI95 [27, 45]. The majority of children with rotavirus infection were unvaccinated (36 cases) and had watery diarrhoea (47 cases), of high frequency per day/per admission 9.6 ± 3.4 and accompanied by severe dehydration (30 cases). A statistically significant difference in mean Vesikari score was observed between unvaccinated and vaccinated children (12.7 vs 10.7 *p*-value 0.024).

**Conclusion:**

Rotavirus infection in hospitalized children under 5 years of age is characterized by a severe clinical manifestation. Epidemiological surveillance is needed to identify risk factors associated with the infection.

## Background

For several decades, acute diarrhoea has been a major cause of morbidity and mortality in children under 5 years of age worldwide, especially in resource-limited settings such as sub-Saharan Africa (SSA) [1, 2]. Rotavirus is the main causative agent of acute diarrhoea in children under 5 years of age. It is estimated to be responsible for more than 258 million annual episodes of diarrhoea worldwide, causing up to 128,500 deaths per year in children under five, the majority of whom are in SSA [2, 3]. Regardless of where they live, almost all children are infected with rotavirus at least once before the age of 5 years, despite efforts to improve immunisation, hygiene and access to safe water in many parts of the world [4, 5]. In the Democratic Republic of the Congo, rotavirus infection is a real public health problem and before the introduction of the rotavirus vaccine, the rotavirus mortality rate was estimated to be around 7% of the global under-five mortality rate in DRC [3, 6]. The clinical manifestations of rotavirus infection vary greatly with age and previous exposure to the virus, ranging from asymptomatic infection to severe manifestations of diarrhoea, vomiting and dehydration [5, 7]. The assessment of the severity of rotavirus infection by the Vesikari score has been proposed by several authors and to date it is one of the means of assessing the impact of vaccination on severe disease [8–10]. Several studies have demonstrated the change in the epidemiological and clinical profile of rotavirus infection in some parts of the world since the introduction of different rotavirus vaccines [9–11]. Presented as the best way to protect children against rotavirus in several countries [11, 12]; it is since 2019, the DRC, with the support of the World Health Organisation (WHO), has introduced the Rotasiil vaccine into the routine vaccination of children, following the example of other countries in Africa and this vaccine was recommended for all children in the DRC within the framework of the National Expanded Programme on Immunisation [13, 14]. This vaccine, manufactured by the Serum Institute of India, includes five genotypes G1, G2, G3, G4 and G9 that are frequently involved in acute diarrhoea in children in Africa [6, 15]. Although data on the impact of vaccination on the epidemiological and clinical changes of rotavirus infection in children under 5 years of age are available globally and in some SSA countries [7, 10], there are still few data available for the DRC, especially the city of Kisangani. The objective of this study was to describe the clinical characteristics of children with acute rotavirus diarrhoea after the introduction of Rotasiil in the DRC.

## Methods

### Type and framework of the study

We conducted a cross-sectional study of acute diarrhoea in children under 5 years of age admitted to 4 hospitals in Kisangani, DRC. These were children for whom acute diarrhoea was the main reason for admission to these hospitals. We conveniently chose four research sites in the city of Kisangani: the Department of Paediatrics of the Cliniques Universitaires, a tertiary-level state hospital; the Hôpital Général de Référence de Makiso-Kisangani, a secondary-level state hospital; the Centre de Santé Alabul, a private hospital; and the Nouveau Village de Pédiatrie, a private hospital.

### Study sample

We included children under 5 years of age with diarrhoea admitted for care at the research sites from 01 May to 31 October 2022. We performed exhaustive sampling according to the following inclusion criteria: age ≤ 5 years, acute diarrhoea, maternal consent to participate in the study. Non-inclusion criteria were neonate, a history of chronic diarrhoea or any patient admitted for another medical reason who also has diarrhea and maternal non-consent to study participation. We first compared children with rotavirus infection with children without rotavirus infection and then we compared children with unvaccinated rotavirus with children with vaccinated rotavirus. The calculation of the number of cases necessary to allow comparison between the groups according to the Vesikari score was carried out with the R software, taking into account an alpha of 0.05, a power of 95% of the test, a delta value of 2, a standard deviation of 3 and a ratio of 1. We obtained the minimum expected number of cases of 59.

### The course of the study

Using a pre-designed form, we prospectively collected data from children under 5 years of age for whom diarrhoea was the main admission diagnosis. Each patient was examined by a physician for the diagnosis of diarrhoea. Epidemiological and clinical variables of interest were obtained by interviewing the mother and clinical examination of the child. These included age, sex, vaccination status, mother’s education level, marital status, mother’s occupation, mother’s type of hygienic latrine, frequency of diarrhoea per day/per admission, duration of diarrhoea in days, appearance of diarrhoea, degree of dehydration, presence of fever and vomiting. The clinical severity of rotavirus infection was assessed by the Vesikari score (see Table 1). The rotavirus vaccination status was verified from the child’s vaccination card. Data on the management of children with diarrhoea were also collected. After informed consent from the child’s mother, a stool sample was collected and sent to the laboratory for detection of rotavirus and possibly adenovirus.

**Table 1:** Vesikari score

Parameter	1	2	3
Diarrhea			
Maximum number stools per day	1–3	4–5	≥ 6
Diarrhea Duration (day)	1–4	5	≥ 6
Vomiting			
Maximum number per day	1	2–4	≥ 5
Vomiting duration (day)	1	2	3
Maximum body temperature (°C)	37.1–38.4	38.5–38.9	≥ 39.0
Severity of dehydration (%)	N/A	1–5	≥ 6
Treatment	Rehydration	Hospitalization	N/A
Severity rating scales	< 7 (mild)	7–10 (moderate)	11 (severe)

(Adapted from Ruuska T and Vesikari T. Scand J Infect Dis 1990;22:259-674 [8])

### Laboratory analysis or test procedure

**Fig. 1:**
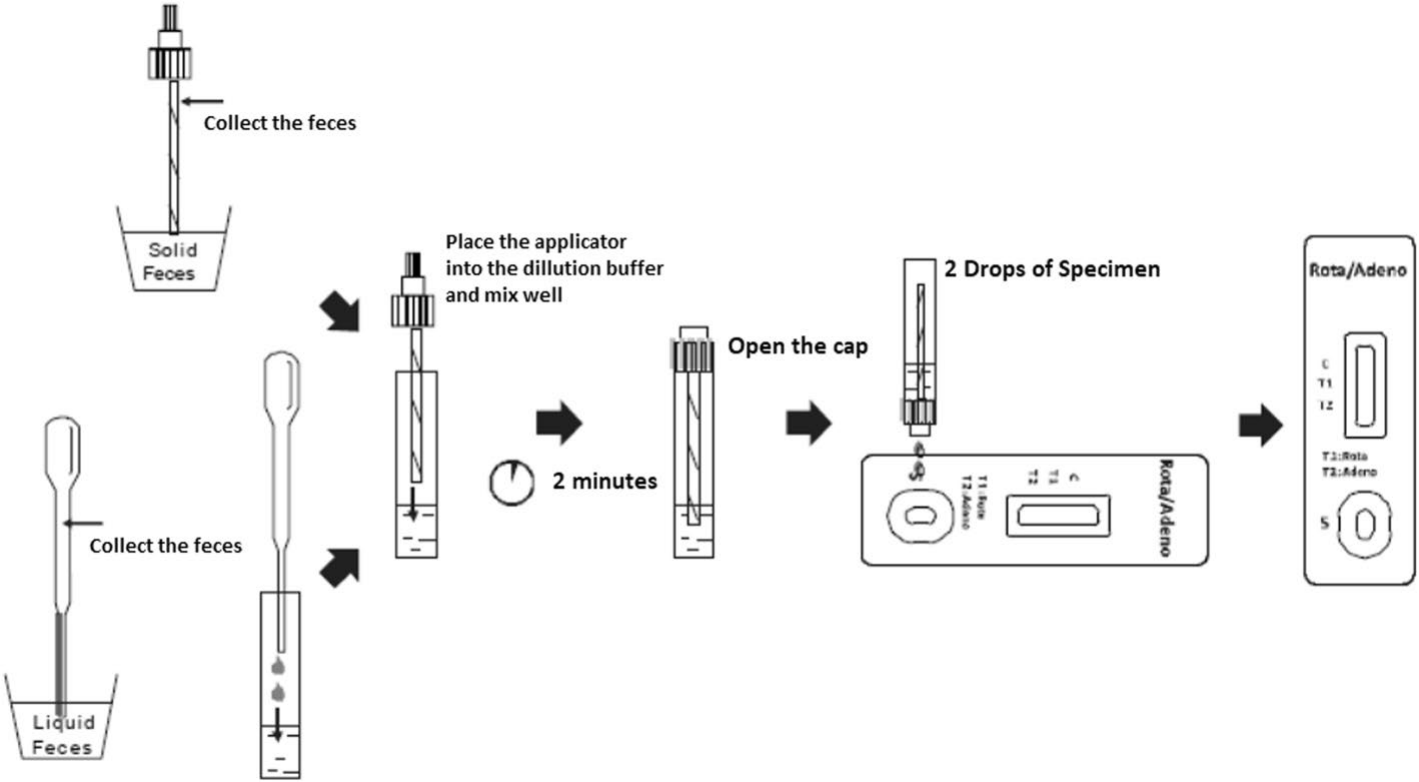
Test procedure [16]

Rotavirus testing was performed on stool samples from the children included in the study by a trained laboratory technician following the standard operating procedure of a validated commercial test (BYOSYNEX adenovirus/rotavirus BSS, France). BYOSYNEX adenovirus/rotavirus BSS is a rapid in vitro diagnostic immuno-chromatographic test for the qualitative detection of rotavirus and adenovirus in human stool samples [16]. The faecal sample was collected (1-2 ml or 1-2 g) in a clean, dry, impermeable tube containing no detergents or preservatives and taken immediately to the laboratory for analysis. Using an applicator 50 μl or 50 mg was transferred to a tube containing extraction buffer and shaken vigorously for 2 minutes before placing 2 full drops of extracted sample (approximately 80 μl) into the sample well of the test cassette (see Fig. 1). The result was read after 10 minutes. The primary endpoint was the presence or absence of rotavirus antigens in the stools of children with acute diarrhoea according to the result. The test also detected Ad40 and Ad41 adenovirus antigens simultaneously. The test result was recorded in the patient’s chart and communicated to the mother in the child’s care.

### Operational definitions

Diarrhoea was defined as passing loose or watery stools 3 or more times per day. Rotavirus diarrhoea was diarrhoea with the detection of rotavirus in the stool. A rotavirus-vaccinated child was one who had received at least one dose of rotavirus vaccine, and a fully vaccinated child was one who had received 3 doses of rotavirus vaccine. A dry toilet is a simple pit latrine that does not use water, while a water toilet is a flush toilet.

### Statistical analysis

The data were recorded in an Excel database and the statistical analysis was carried out with the R software version 4.2.1. Differences between groups were analysed using the Pearson Chi-square test for categorical variables and the Wilcoxon test for continuous variables. Multiple linear regression was performed for factors associated with the clinical severity of acute rotavirus diarrhoea according to the Vesikari score.

### Ethical consideration

The study protocol was approved by the ethics and research committee of the University of Kisangani (CER/013/GEAK/2022), and the provincial health authority of Tshopo Province by letter 701/DPS/TSHOPO/SEC/0359/2022. All samples were collected in a standard of care setting for diagnostic and therapeutic purposes. Data were fully anonymised at the time of collection.

## Results

A total of 178 children under 5 years of age with acute diarrhoea were admitted to the four hospitals during the study period; 4 (2.2%) children were excluded because no consent was provided and 9 (5%) had no stool sample. Therefore, a total of 165 (92.6%) children under 5 years of age with acute diarrhoea were included in the study over a 6-month period at the research sites. Analysis of stool samples for rotavirus and adenovirus antigenic testing revealed 59 cases of rotavirus, 36% CI_95_ [27, 45]; 10 cases of adenovirus, 6.1%; 2 cases of rotavirus and adenovirus co-infection, 1.2%; and 94 negative cases, 57%.

### Socio-demographic characteristics of children under 5 years of age with acute diarrhoea

Table 2 presents the socio-demographic characteristics of children under 5 years old with acute diarrhea in a comparison between children without rotavirus and children with rotavirus.

**Table 2:** Socio-demographic characteristics of children under 5 years of age with rotavirus

Characteristic	Negative, ***N*** = 94^***1***^	Rotavirus, ***N*** = 59^***1***^	***p***-value^***2***^
**Age (months)**			0.3
[1 - 11]	61 (62.9%)	36 (37.1%)	
[12 - 24]	19 (67.9%)	9 (32.1%)	
[25–59]	14 (50.0%)	14 (50.0%)	
**Gender**			0.087
Female	40 (70.2%)	17 (29.8%)	
Male	54 (56.2%)	42 (43.8%)	
**Vaccinated against rotavirus**			**< 0.001**
No	29 (44.6%)	36 (55.4%)	
Yes	65 (73.9%)	23 (26.1%)	
**Number of vaccine doses**			**< 0.001**
0	29 (44.6%)	36 (55.4%)	
1	6 (85.7%)	1 (14.3%)	
2	9 (42.9%)	12 (57.1%)	
3	50 (83.3%)	10 (16.7%)	
**Mother’s occupation**			**0.048**
With employment	30 (62.5%)	18 (37.5%)	
Student	0 (0.0%)	4 (100.0%)	
Unemployed	64 (63.4%)	37 (36.6%)	
**Mother’s level of education**			0.3
Primary	0 (0.0%)	1 (100.0%)	
Secondary	55 (59.1%)	38 (40.9%)	
Academic	39 (66.1%)	20 (33.9%)	
**Marital status**			0.2
Single	3 (33.3%)	6 (66.7%)	
Married	72 (61.5%)	45 (38.5%)	
Free union	19 (70.4%)	8 (29.6%)	
**Household water**			0.6
Well	10 (52.6%)	9 (47.4%)	
Tap	83 (62.9%)	49 (37.1%)	
Source	1 (50.0%)	1 (50.0%)	
**Type of mother’s toilet**			0.8
Water closet	57 (60.6%)	37 (39.4%)	
Dry toilet	37 (62.7%)	22 (37.3%)	
1 n (%) 2 Pearson’s Chi-squared test			

We observed a statistically significant difference between children without rotavirus and children with rotavirus according to their vaccination status (73.9% vs 26.1% for vaccinated children *p*-value < 0.001) and the occupation of their mothers as shown in Table 2. We also observed a statistically significant difference between children without rotavirus infection and children with rotavirus according to the number of vaccine doses received. Children aged 1 to 11 months were in the majority (61 cases, or 62.9% negative vs 36 cases, or 37.1% rotavirus negative) with no statistically significant difference from other age groups. The female/male sex ratio of children with rotavirus was 0.4.

### Clinical profile of children under 5 years of age with acute diarrhoea

In a bivariate analysis, we compared the clinical manifestations observed in children with rotavirus and children who tested negative (see Table 3). These results provide information on the clinical manifestations of children with acute diarrhoea in hospital settings.

**Table 3:** Clinical characteristics of children under 5 years of age with rotavirus

Parameters		Negative *N* = 94^a^	Rotavirus *N* = 59^a^	***p***-value^b^
**Diarrhoea frequency per day/per admission, Mean (SD)**		5.9 (2.2)	9.6 (3.4)	**< 0.001**
**Duration of diarrhea in days, Mean (SD)**		3.6 (1.4)	6.1 (2.0)	**< 0.001**
**Aspects**				**< 0.001**
	Mucous	56 (82.3%)	12 (17.7%)	
	Aqueous	37 (44.0%)	47 (56.0%)	
	Bloody	1 (100.0%)	0 (0.0%)	
**Vomiting**				**0.002**
	No	30 (83.3%)	6 (16.7%)	
	Yes	64 (54.7%)	53 (45.3%)	
**Dehydration**				**< 0.001**
	Slight	9 (100.0%)	0 (0.0%)	
	Moderate	65 (69.1%)	29 (30.9%)	
	Severe	20 (40.0%)	30 (60.0%)	
**Fever**				**< 0,001**
		35 (87.5%)	5 (12.5%)	
		59 (52.2%)	54 (47.8%)	
**Cough**				0.4
	No	40 (58.0%)	29 (42.0%)	
	Yes	54 (64.3%)	30 (35.7%)	
**Abdominal pain**				**< 0,001**
	No	66 (86.8%)	10 (13.2%)	
	Yes	28 (36.4%)	49 (63.6%)	
**Vesikari Score**				**< 0.001**
	< 7	27 (87.1%)	4 (12.9%)	
	[3 - 7]	45 (88.2%)	6 (11.8%)	
	≥11	22 (31.0%)	49 (69.0%)	
**Vesikari score, Mean (SD)**		9.4 (3.2)	13.1 (2.8)	**0.002**

^a^n (%)

^b^Wilcoxon test; Chi-square test

We observed that the mean frequency per day/per admission of diarrhoea (5.9 vs. 9.3) and the mean duration in days (3.6 vs. 6.1) of diarrhoea were statistically different between the two groups, as were the appearance of the stools (mucusy 82.3% vs. 17.7%, watery 44% vs. 56% *p* value < 0.001) and the degree of severe dehydration (40% vs. 60% *p* value < 0.001) between children without rotavirus infection and children with rotavirus. Vomiting and abdominal pain were also observed in statistically different proportions between children testing negative and those with rotavirus; this was not the case with children with cough. When assessing the clinical severity between the two groups, we observed that the mean score of the children with rotavirus was high and statistically different from the children tested negative.

### Clinical characteristics of vaccinated and unvaccinated children with rotavirus

In Table 4, we compared the clinical characteristics of vaccinated and unvaccinated children with rotavirus infection. No statistically significant differences were observed in the mean frequency of diarrhoea per day/per admission (8.83 vs 9.04 *p*-value 0.3) and the mean duration in days of diarrhoea (6.11 vs 6.17 *p*-value 0.5). While most unvaccinated children had watery diarrhoea compared to vaccinated children (53.2 vs 46.8 *p*-value 0.019). Other clinical parameters (vomiting, dehydration, fever, cough, abdominal pain) did not show statistically significant differences between vaccinated and unvaccinated children with rotavirus infection.

**Table 4:** Clinical characteristics of vaccinated and unvaccinated children with rotavirus

Parameters		unvaccinated *n* = 36^a^	Vaccinated *n* = 23^a^	***p***-value^b^
**Diarrhoea frequency per day/admission, Mean (SD)**		8.83 (3.44)	9.04 (2.03)	0.3
**Duration of diarrhoea in days, Mean (SD)**		6.11 (2.19)	6.17 (1.72)	0.5
**Aspects**				**0.019**
	Glaireux	11 (91.7%)	1 (8.3%)	
	Aqueous	25 (53.2%)	22 (46.8%)	
**Vomiting**				0.4
	No	5 (83.3%)	1 (16.7%)	
	Yes	31 (58.5%)	22 (41.5%)	
**Dehydration**				0.7
	Moderate	17 (58.6%)	12 (41.4%)	
	Severe	19 (63.3%)	11 (36.7%)	
**Fever**				0,15
	No	5 (100.0%)	0 (0.0%)	
	Yes	31 (57.4%)	23 (42.6%)	
**Cough**				0.5
	No	19 (65.5%)	10 (34.5%)	
	Yes	17 (56.7%)	13 (43.3%)	
**Abdominal pain**				0,5
	No	5 (50.0%)	5 (50.0%)	
	Yes	31 (63.3%)	18 (36.7%)	
**Vesikari Score**				0.2
	< 7	4 (50.0%)	4 (50.0%)	
	[7 - 10]	5 (41.7%)	7 (58.3%)	
	≥11	27 (69.2%)	12 (30.8%)	
**Vesikari score, Mean (SD)**		12.7 (3.2)	10.7 (2.8)	**0.024**

^a^n (%)

^b^Wilcoxon test; Fisher’s test, Pearson’s Chi-square test

When assessing the clinical severity of rotavirus infection between vaccinated and unvaccinated children according to the Vesikari score, it was found that the mean score in unvaccinated children was high compared to vaccinated children (12.7 vs 10.7 *p*-value 0.024). However, no statistically significant difference was observed for the different classes of the Vesikari score.

### Multivariate analysis of factors associated with clinical severity of acute rotavirus diarrhea in children under 5 years of age according to the Vesikari score

The Vesikari score was used as a means of assessing the clinical severity of rotavirus infection. In a multiple linear regression analysis, we studied the variation of the score according to some socio-demographic factors. The age group 24–59 months and unemployed mothers had a statistically significant variation in the Vesikari score (see Table 4).

We observed in a multivariate analysis by linear regression in Table 5, that children aged 24–59 months were associated with a Vesikari score of minus 3.7 or minus 4 compared to children in the 1–11 month age group, whereas no statistically different association was observed with children aged 12–23 months. Vaccination status was not statically associated with a change in the Vesikari score, nor was the mother’s education level or the different types of household water. However, we observed an association with a score of minus 2.8 or minus 3 for mothers of unemployed patients compared to mothers of employed patients.

**Table 5:** multiple linear regression of factors associated with the Vesikari score of rotavirus diarrhea

Coefficients:	Estimate Std.	Error	t value	***P*** value
Age [12–23 months]	−0.2919	0.8182	−0.357	0.723
[24–59 months]	−3.7355	0.8662	−4.313	**8.21e-05** ***
Gender Male	− 0.4845	0.7316	−0.662	0.511
Vaccinated with Rotasiil YES	0.7561	0.6704	1.128	0.265
Mother’s level of education Secondary	−0.2876	2.2831	−0.126	0.900
Mother with university education	0.2017	2.4454	0.082	0.935
Mother Student	−0.2237	1.3755	−0.163	0.872
Mother Unemployed	−2.882	0.6535	−4.410	**5.99e-05** ***
Household water: Tap	1.1934	0.873	1.367	0.178
Household water: Source	3.8031	2.3811	1.597	0.117
Dry pit toilet	0.8629	0.6588	1.31	0.197

Significance of codes: ‘***’ 0.001

Residual standard error: 2.093 on 47 degrees of freedom

Multiple R-squared: 0.5553, Adjusted R-squared: 0.4513

F-statistic: 5.336, *p*-value: 2.044e-05

## Discussion

The aim of this study was to describe the clinical characteristics of children under 5 years of age with acute diarrhoea after the introduction of rotavirus vaccination of children in Kisangani city hospitals. The hospital prevalence of rotavirus acute diarrhoea in children under 5 years was 36% CI_95_ [27, 45]. Prior to the introduction of vaccination, Heylen E and Bibi found a prevalence ranging from 18.8 to 37.1% with an average of 29% in children under 5 years of age [17]. However, in Lubumbashi, Sangaji found a high prevalence of 53.9% in children under 5 years of age suffering from acute diarrhoea [18], while in sentinel sites, the prevalence of acute rotavirus diarrhoea in children under 5 years of age was 60% [6]. We believe that the high proportion of unvaccinated children could explain this prevalence. Looking at the socio-demographic characteristics of children with acute rotavirus diarrhoea, we noted that children in the 1–11 month age group were the most affected in 36 out of 59 cases. In Kenya Moendo, observed that the prevalence of rotavirus diarrhoea was high in children aged 17–24 months [10], while in the Sangaji study it was children under 12 months who were most affected [18], although in Rwanda Claudine showed that the risk of acute diarrhoea in general was highest in children aged 12–23 months [19]. The female/male sex ratio was 0.4 in contrast to other studies in Lubumbashi where the sex ratio was about 1. We noted that the proportion of children vaccinated against rotavirus was lower in children with rotavirus than in children without rotavirus (73.9% negative vs 26.1% rotavirus), Zakaret also found a low proportion of 23.9% in his study [20]. This is in contrast to the Moendo study in Kenya where almost all children were vaccinated [10]. This situation could be explained by the low promotion of the rotavirus vaccine in Kisangani and context of the Covid 19 pandemic, despite the fact that this vaccine is recommended for all children according to the National Expanded Programme on Immunisation in the DRC and that this vaccine would contribute to the protection of vaccinated children, as already demonstrated elsewhere by other authors [9, 10, 20, 21]. We noted that the mothers of children with rotavirus diarrhoea had in most cases a secondary level of education (55 cases, i.e. 59.1% negative vs. 38 cases, i.e. 40.9% rotavirus) and were mostly unemployed (64 cases, i.e. 63.4% negative vs. 37 cases, i.e. 36.6% rotavirus); they used tap water in their households (83 cases, i.e. 62.9% negative vs. 49 cases, i.e. 37.1% rotavirus), although the difference was not statistically significant.

Clinically, in a bivariate analysis between the two groups, we observed that there was a statistically significant difference in the mean frequency per day/per admission of diarrhoea (5.9 vs 9.6) and the mean duration in days of rotavirus diarrhoea (3.6 vs 6.1). The same was true of the proportions of vomiting (54.7% vs. 45.3%) and dehydration in their various degrees. In this study, it was noted that the mean clinical severity score of Vesikari was higher in children with rotavirus with a statistically significant difference (9.4 vs 13.1) and by comparing the different categories, we observed that 69% of the rotavirus infection cases were severe contrary to Moendo who reported 28.3% of the severe cases and about 39.6% of the mild cases [10]. Comparing the clinical characteristics of rotavirus infection between vaccinated and unvaccinated children, we observed a higher score in unvaccinated children than in vaccinated children with a statistically significant difference (12.7 vs 10.7 *p*-value 0.024). Studies have shown that vaccination protects children against severe forms of rotavirus infection [15, 22] and we believe this would explain the difference in Vesikari score between vaccinated and unvaccinated children. In Lebanon, Zakaret reported a mean score of 10.9 ± 1.7, closer to Shim Dong who found a mean score of 10 ± 3.3 in a study evaluating the predictive value of this sore between viral and bacterial causes of paediatric gastroenteritis [20, 22, 23]. Our observation could be explained by the fact that these are severe cases that frequent hospital facilities more often, although some authors think that the performance of this score should be improved [24]. Cough as a respiratory symptom was observed between the two groups with no statistically significant difference (64.3% vs 35.7% *p* value 0.4). Apart from clinical intestinal manifestations, cough or convulsions have been described during rotavirus infection in children by other authors [25], whereas in this study cough was observed without significant difference from children without rotavirus infection. In a multiple linear regression analysis, we looked for factors that could influence the Vesikari score in children with rotavirus infection (see Table 5); the results of this analysis showed that the 24–59 month age range of children with rotavirus infection was associated with a clinical severity score of approximately minus 4 while it was associated with minus 3 for mothers unemployed with a statistically significant difference. The high incidence of rotavirus infection in children under 2 years of age has been reported in several studies and this is the period when primary rotavirus infection is usually observed [18, 26, 27], this would explain the frequency of severe clinical forms compared to children over 2 years of age and we don’t have an explanation for the relationship with unemployed mothers. Finally, we believe that the interpretation of the results of this study must be made taking into account the limitations of the sample size and the rotavirus diagnostic method we used. However, this study provides important and reliable information in these four hospitals where rotavirus screening is not part of routine hospital care.

## Conclusion

This study showed that rotavirus infection in the hospital setting after the introduction of the rotavirus vaccine in Kisangani is characterised by severe clinical manifestations of high frequency watery diarrhoea accompanied by severe dehydration and affects in the majority of cases unvaccinated children in the age group of 1 to 11 months. These results highlight the burden of rotavirus-associated diarrhoea in children under 5 years of age in Kisangani, Democratic Republic of the Congo. Surveillance studies are needed to identify risk factors associated with rotavirus infection.

## Data Availability

The datasets used in this study are available from the corresponding author upon reasonable request.

## Abbreviations

SSA: Sub-Saharan Africa
CI: Confidence interval
WHO: World Health Organization
DRC: Democratic Republic of the Congo
vs: Versus
Std: Standard
SD: Standard deviation
